# Oral Adverse Reactions Caused by Over-the-Counter Oral Agents

**DOI:** 10.1155/2015/196292

**Published:** 2015-03-26

**Authors:** Vanja Vucicevic Boras, Vlaho Brailo, Ana Andabak Rogulj, Danica Vidovic Juras, Dragana Gabric, Danko Velimir Vrdoljak

**Affiliations:** ^1^Department of Oral Medicine, School of Dental Medicine, Croatia University of Zagreb and Clinical Hospital Centre Zagreb, Kispaticeva 12, Croatia; ^2^Department of Oral Medicine, School of Dental Medicine, University of Zagreb, Croatia; ^3^Clinical Hospital Centre Zagreb, Kispaticeva 12, Croatia; ^4^Department of Oral Surgery, School of Dental Medicine, University of Zagreb, Croatia; ^5^Clinic for Tumours, Clinical Hospital Centre Sisters of Mercy, Zagreb, Croatia

## Abstract

Over-the-counter products rarely cause unwanted reactions in the oral cavity. Oral reactions to these agents are not specific and might present with various clinical oral findings. Detailed medical history is a key to the proper diagnosis of these lesions and fortunately other diagnostic procedures are rarely needed. Lesions are usually managed with elimination of the offending agent and with topical steroids. In more severe cases systemic steroids should be applied.

## 1. Introduction

Oral lesions due to the over-the-counter (OTC) products for oral cavity are rare. Such lesions may be seen as various different clinical forms mimicking other oral well-known diseases. Injuries of the oral mucosa might develop after direct contact with chemical agents. Clinical presentation of these lesions might differ according to the composition, pH, and concentration of the chemical agent(s), the quantity applied, the manner and duration of tissue contact, the extent of penetration into tissue, and the mechanism of action. In the oral cavity, chemical substances cause diffuse erosive lesions ranging from simple desquamation (mucosal sloughing) to complete mucosal detachment with extension into the submucosa [1]. Clinical diagnosis of a chemical burn of the oral mucous membranes may be a diagnostic challenge and a detailed history and review of a patient's medical condition will help to differentiate possible causes of the presenting lesion(s). The challenge is to obtain relevant information such as the temporal relationship between the OTC product use and onset of oral lesions. Removal of the agent is critical to ensure healing. This paper's intent is to illustrate the appearance of an OTC induced oral lesion caused by oral agents [2].

## 2. Case Reports

**Figure 1:**
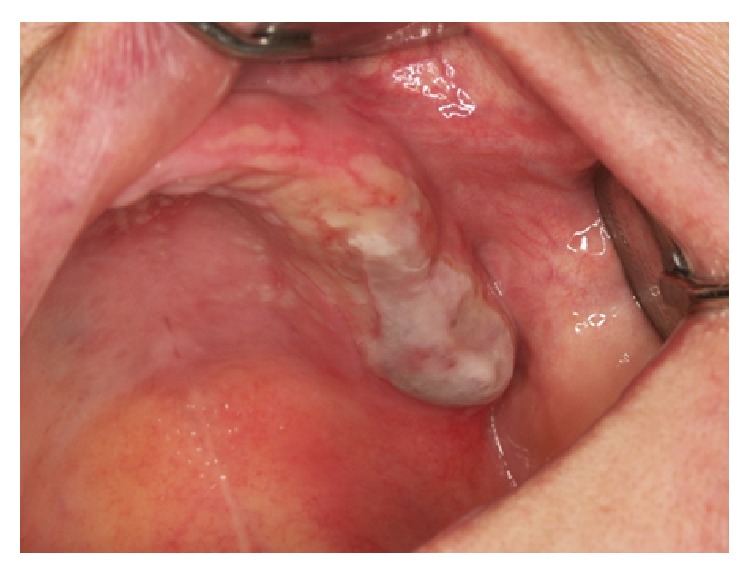
Oral lesion caused by* Tinctura adstringens.*

**Figure 2:**
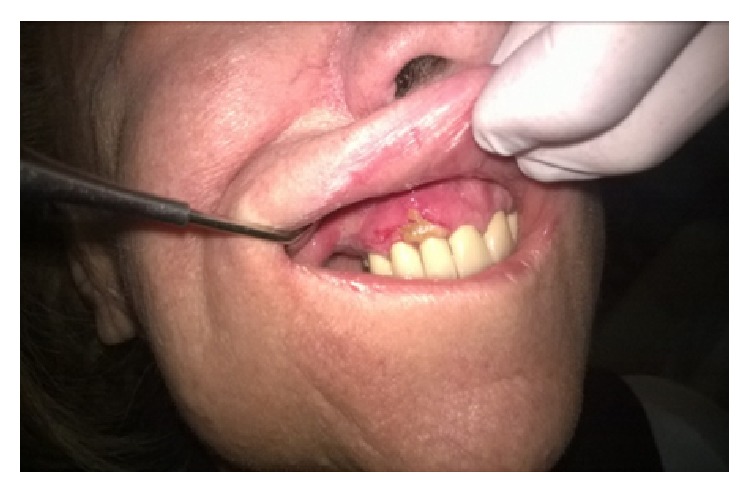
Propolis induced oral lesions.

**Figure 3:**
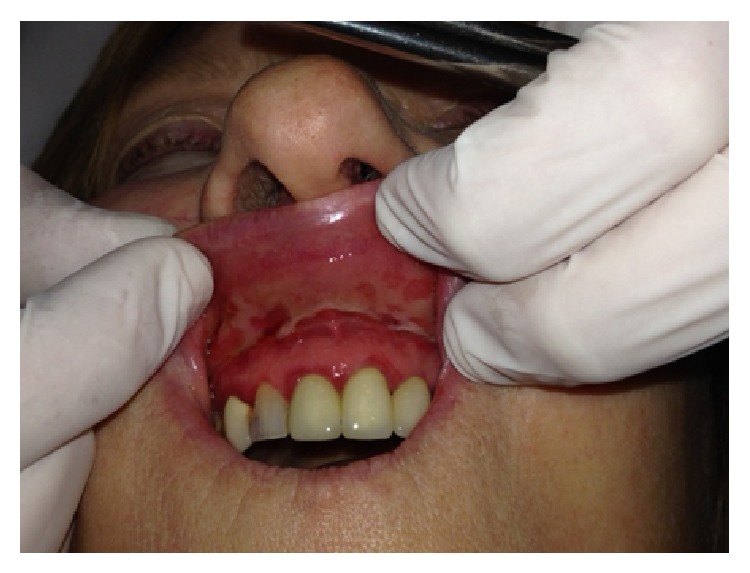
Gentian violet induced oral lesions.

**Figure 4:**
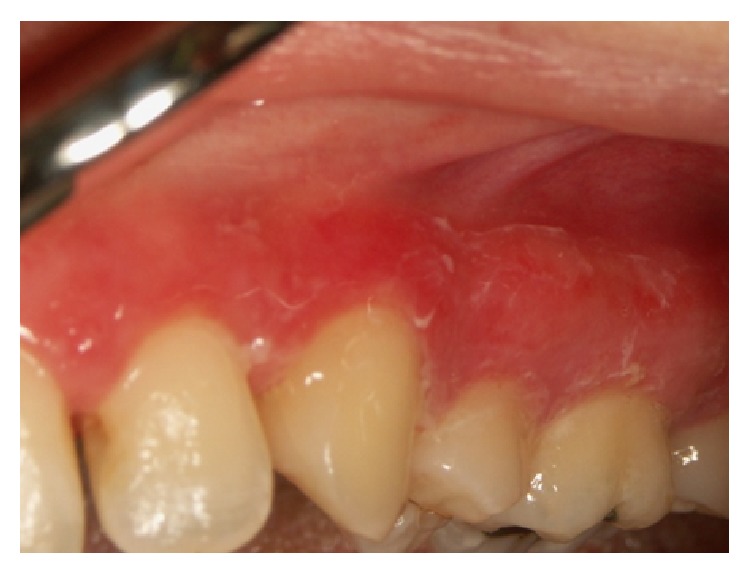
Sloughing of the upper gingiva due to the 0.2% chlorhexidine digluconate.

**Figure 5:**
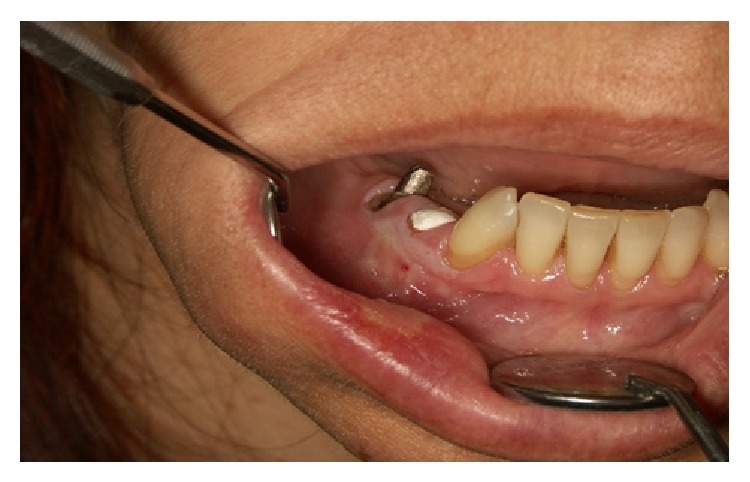
Chemical burn of the gingiva and labial lower mucosa caused by sodium hypochlorite.

1. Certain magistral preparations (i.e., made in pharmacy) such as* Tinctura adstringens* (TA) which are usually dissolved in alcohol solution might lead to the oral mucosal damage (Figure 1). Patient had painful sensation on the gingiva on left part of the upper jaw and treated the affected area with TA. The lesions developed two days after he started to use the solution. He was advised to immediately stop using TA and was given corticosteroid in orabase (betamethasone) to be applied three times a day. After 5 days he was free of oral lesions.
2. Patient who applied propolis (or bee glue is a resinous mixture that* honey bees* collect from tree buds, sap flows, or other botanical sources) in alcohol solution for the gingival inflammation which resulted probably in chemical burn due to the alcohol content within the propolis spray (Figure 2). Patient otherwise suffers from hypertension and gastric disturbances. She was advised to immediately stop using propolis and was given corticosteroid in orabase (betamethasone) to be applied three times a day. After 3 days she was free of oral lesions.
3. Patient applied gentian violet (GV) solution on the inflamed gingiva which led to the exfoliative lesions (Figure 3). Patient detailed medical history revealed that she suffers from sinusitis and hypothyroid disease. She was advised to immediately stop using GV and was given corticosteroid in orabase (betamethasone) to be applied three times a day. Furthermore, she was given subcutaneous corticosteroid injection Depo Medrol (methylprednisolone acetate) and after 7 days she was free of oral lesions.
4. Patient was using mouthwash containing 0.2% chlorhexidine digluconate in a regularly prescribed manner; however, sloughing of the oral mucosa developed (Figure 4). The patient was otherwise healthy. He was advised to immediately stop using mouthwash. No other therapy was given as the patient was not in pain.
5. Dentist was performing root canal treatment which resulted in accidental chemical burn of the gingiva and labial lower mucosa caused by sodium hypochlorite (Figure 5). The patient was otherwise healthy. Patient was given corticosteroid in orabase (betamethasone) to be applied three times a day and the lesions subsided after 4 days.

## 3. Discussion

Certain magistral preparations (i.e., made in pharmacy) such as* Tinctura adstringens* (TA) are sold by pharmacists. This solutions content is alcohol and certain herbs known to have astringent action supposing its anti-inflammatory role in gingival inflammation. These products are usually sold by pharmacists when patients seek help for their oral condition without taking advice of their dentists.

In Croatia, the use of propolis for treatment of various oral conditions is quite popular although it is known that it can lead to adverse effects in the oral cavity. It is mainly recommended by pharmacists. Propolis induced oral lesions most often present as diffuse oral erosions that can affect all regions of oral cavity, that is, parts of the mucosa where propolis was used (Figure 2) [3].

Although gentian violet (GV) is widely used for skin and/or mucosal disinfection, in some circumstances, GV might lead to the unwanted side effects [4, 5]. Nyst et al. [6] reported that side effects of GV were local irritation and ulceration which were infrequent and reversible. Participants who received GV solutions at 0.0085% and 0.1% reported a bitter taste (6 out of 15) which resolved upon treatment discontinuation; however, no side effects were recorded at GV concentration of 0.00165%. Earlier this year we reported the case of the patient who developed oral lesions after a GV use two times a day (Figure 3) [7]. This case report certainly shows that in susceptible patients, regular application might lead to the development of unwanted side effects.

Oral health care products prescribed usually for various inflammatory oral conditions have been recognized as possible causes of different oral lesions such as sloughing of the oral mucosa (Figure 4), desquamative gingivitis, gingival ulcerations, and fixed drug eruptions. Various adverse reactions including anaphylactic shock have already been reported after the topical application of chlorhexidine. Moghadam et al. [8] reported a patient with the reaction in the form of fixed drug eruption after the use of a mouthwash containing chlorhexidine. Kuttan et al. [9] reported a case of severe mucosal injuries following misuse of an undiluted over-the-counter mouthwash with a high alcohol content (70%), oil of peppermint, and arnica. Murdoch-Kinch et al. [10] reported a case of chemical injury to the oral mucosa that resulted in obstructive sialadenitis of the submandibular glands. The injury occurred when a patient chewed, swished, and expectorated an immersion-type denture-cleansing tablet attempting to clean a fixed bridge. Touyz and Hille [11] reported a case of an unusual chemical burn, confined to the masticatory mucosa, produced by abusive fresh fruit ingestion and concomitant excessive use of mouthwashes.

There are several reports within dental literature upon chemical burns as a result of 3% hydrogen peroxide misuse [12, 13]. Nowadays hydrogen peroxide is used mainly in endodontics for root canal irrigation. Rostami and Brooks [12] reported that injudicious use (more than two minutes) of over-the-counter 3% hydrogen peroxide can result in a painful chemical burn of the sublingual, buccal mucosa, and gingiva. According to Rees and Orth [14] improper use of H_2_O_2_ at a concentration greater than 3% can lead to epithelial necrosis.

The diagnosis of an OTC induced oral lesion is established upon clinical examination and detailed patient's history with special emphasis on the temporal relationship between OTC use and the onset of the lesions according to the Naranjo ADR Probability Scale [15]. The scale consists of 10 differently scored questions about the adverse event. A final score provides indication of the overall probability that the adverse event represents an adverse reaction to an OTC. Additional tests like histopathological evaluation or direct immunofluorescence can be performed in order to exclude known oral diseases.

The treatment of chemical burns due to the OTC products consists of irrigation to minimize the product effect and local steroid therapy. Baruchin et al. [16] reported that a protective emollient agent such as a film of methyl cellulose may provide relief. In cases where severe pain is present, local anaesthetic gel can be included. Our opinion is that elimination of the offending agent and local steroid therapy (betamethasone in orabase to be applied three times a day) is sufficient therapy in most cases. However if the lesions are more pronounced and disable patient to eat, systemic corticosteroid therapy should be given.
