# Corrigendum to “Oral Adverse Reactions Caused by Over-the-Counter Oral Agents”

**DOI:** 10.1155/2019/2816785

**Published:** 2019-11-21

**Authors:** Vanja Vucicevic Boras, Vlaho Brailo, Ana Andabak Rogulj, Danica Vidovic Juras, Dragana Gabric, Danko Velimir Vrdoljak

**Affiliations:** ^1^Department of Oral Medicine, School of Dental Medicine, Croatia University of Zagreb and Clinical Hospital Centre Zagreb, Kispaticeva 12, Croatia; ^2^Department of Oral Medicine, School of Dental Medicine, University of Zagreb, Croatia; ^3^Clinical Hospital Centre Zagreb, Kispaticeva 12, Croatia; ^4^Department of Oral Surgery, School of Dental Medicine, University of Zagreb, Croatia; ^5^Clinic for Tumours, Clinical Hospital Centre Sisters of Mercy, Zagreb, Croatia

In the article titled “Oral Adverse Reactions Caused by Over-the-Counter Oral Agents” [1], there is a mismatch between the text and the cited references [8] and [9] that should be corrected as follows:

Reference [8] should be replaced with the following reference: Moghadam BKH, Drisko CL, Gier RE. “Chlorhexidine mouthwash-induced fixed drug eruption: case report and review of the literature.” Oral Surc Oral Med Oral Pathol 1991; 71 : 431-4 [2].

Reference [9] should be replaced with the following reference, which was previously added as reference [8]: B. K. H. Moghadam, R. Gier, and T. Thurlow, “Extensive oral mucosal ulcerations caused by misuse of a commercial mouthwash,” Cutis, vol. 64, no. 2, pp. 131–134, 1999 [3].

Accordingly, the following statement in Discussion citing reference [9] should be changed from “Kuttan et al. [9] reported a case of severe mucosal injuries following misuse of an undiluted over-the-counter mouthwash with a high alcohol content (70%), oil of peppermint, and arnica.” to “Moghadam et al. [9] reported a case of severe mucosal injuries following misuse of an undiluted over-the-counter mouthwash with a high alcohol content (70%), oil of peppermint, and arnica.”
